# Aboriginal community-controlled aged care: principles, practices and actions to integrate with primary health care

**DOI:** 10.1017/S1463423621000542

**Published:** 2021-10-07

**Authors:** Anna Dawson, Stephen Harfield, Carol Davy, Anna Baker, Elaine Kite, Graham Aitken, Kim Morey, Annette Braunack-Mayer, Alex Brown

**Affiliations:** 1 Wardliparingga Aboriginal Health Equity, South Australian Health and Medical Research Institute, Adelaide, Australia; 2 The Society of Saint Hilarion, Adelaide, Australia; 3 Institute for Urban Indigenous Health, Brisbane, Australia; 4 Aboriginal Community Services, Adelaide, South Australia; 5 School of Health and Society, University of Wollongong, Wollongong, Australia; 6 Aboriginal Research Unit, Adelaide Medical School, Faculty of Health and Medical Sciences, The University of Adelaide, Adelaide, South Australia

**Keywords:** Aboriginal and Torres Strait Islander, Aboriginal community control, aged care, Indigenous

## Abstract

**Aim:**

To identify: 1) best practice aged care principles and practices for Aboriginal and Torres Strait Islander older peoples, and 2) actions to integrate aged care services with Aboriginal community-controlled primary health care.

**Background:**

There is a growing number of older Aboriginal and Torres Strait Islander peoples and an unmet demand for accessible, culturally safe aged care services. The principles and features of aged care service delivery designed to meet the unique needs of Aboriginal and Torres Strait Islander peoples have not been extensively explored and must be understood to inform aged care policy and primary health care planning into the future.

**Methods:**

The research was governed by leaders from across the Aboriginal community-controlled primary health care sector who identified exemplar services to explore best practice in culturally aligned aged care. In-depth case studies were undertaken with two metropolitan Aboriginal community-controlled services. We conducted semi-structured interviews and yarning circles with 46 staff members to explore key principles, ways of working, enablers and challenges for aged care service provision. A framework approach to thematic analysis was undertaken with emergent findings reviewed and refined by participating services and the governance panel to incorporate national perspectives.

**Findings:**

A range of principles guided Aboriginal community-controlled aged care service delivery, such as supporting Aboriginal and Torres Strait Islander identity, connection with elders and communities and respect for self-determination. Strong governance, effective leadership and partnerships, Aboriginal and Torres Strait Islander workforce and culturally safe non-Indigenous workforce were among the identified enablers of aged care. Nine implementation actions guided the integration of aged care with primary health care service delivery. Funding limitations, workforce shortages, change management processes and difficulties with navigating the aged care system were among the reported challenges. These findings contribute to an evidence base regarding accessible, integrated, culturally safe aged care services tailored to the needs of Aboriginal and Torres Strait Islander peoples.

## Introduction

The life expectancy of Aboriginal and Torres Strait Islander (hereafter, respectfully, Indigenous) peoples is improving in Australia, resulting in growing numbers of elders (AIHW, 2015). While the total Indigenous population is projected to increase by 59% between 2011 and 2031, numbers of older Indigenous peoples (≥65 years) are projected to grow by 200% (Biddle, 2013). In mid-2018, it was estimated that there were already more than 123 000 Indigenous peoples aged 50 years or over (AIHW, 2018a) and recent data show that there are 120 aged care organisations across Australia that provide home care to a client base comprising at least 50% Indigenous peoples (AIHW, 2020).

Indigenous elders ‘play a vital role in sustaining strong cultural practices and traditions within their communities with important roles and responsibilities such as passing on knowledge, languages and customs, participating in decision-making ceremonies and looking after country’ (Commonwealth of Australia, 2013: 38). This is despite burdensome health and social challenges: compared with non-Indigenous Australians, older Indigenous peoples experience greater comorbidity, disability, racism, financial insecurity, homelessness and challenges to health and social services’ access (AIHW, 2019). An estimated 13 800 (14%) of older Indigenous peoples (≥50 years) in 2014–15 were part of the ‘Stolen Generations’ (AIHW, 2018b) who, as children in the 20th century, were forcibly removed from their families under successive government policies (Human Rights and Equal Opportunity Commission, 1997). Compared with Indigenous elders who were not removed, members of the Stolen Generations experience double the rates of poor health and social outcomes (AIHW, 2018b).

Clearly, Indigenous-led, culturally safe aged care services are desperately needed to meet the unique needs of older Indigenous peoples and promote and safeguard elder well-being. Aboriginal and Torres Strait Islander elders have reported feeling unwelcome in mainstream organisations where non-Indigenous people have little understanding or respect for their culture (Ranzijn *et al.*, 2010). Experiences of unfair treatment (e.g. racist comments, being ignored or served last when accessing services) is reported by an alarming 31% of older (≥45 years) Indigenous peoples in Australia, and 15% of these report avoiding situations, such as health and government services, as a result (Temple *et al.*, 2019). Indigenous elders in Canada similarly report racism and discrimination as barriers to service access (Schill *et al.*, 2019). Of paramount importance, then, is the availability of culturally safe aged care and primary health care services delivered by trustworthy providers. International evidence including studies from the United States, Canada, Alaska, Australia and Brazil demonstrate that primary health care and aged care services promote the well-being of older First Nations’ peoples through culturally safe care, maintaining Indigenous identity and promoting independence (Davy *et al.*, 2016).

Over the last decade, the Australian aged care system has undergone extensive scrutiny and reform in response to sector-wide challenges identified in the report ‘*Caring for Older Australians*’ (Productivity Commission, 2011). The 2013 *Living Longer Living Better* reforms package provided $3.7 billion of funding (Department of Health, 2012) and an *Aged Care Roadmap* detailed short, medium and long term plans for a more equitable and consumer-led aged care sector (Aged Care Sector Committee, 2016). The recent *Royal Commission into Aged Care Quality and Safety* identified widespread neglect across the aged care system. For Indigenous peoples, it recommended: ‘integrating aged care with other services, such as primary health, mental health and disability services, including services provided by Aboriginal Community Controlled Health Organisations and other existing Aboriginal health and community organisations’ (Commonwealth of Australia, 2019: 190).

Aboriginal Community Controlled Health Organisations (ACCHOs) are ‘a primary health care service initiated and operated by the local Aboriginal community to deliver holistic, comprehensive, and culturally appropriate health care to the community which controls it, through a locally elected Board of Management’ (National Aboriginal Community Controlled Health Organisation No date). There are 143 ACCHOs across Australia, with services in all states and territories (National Aboriginal Community Controlled Health Organisation No date). All ACCHOs provide holistic primary health care services to older Indigenous peoples though few are formally funded to provide aged care services such as home care, respite care and residential aged care. In contrast, there are Aboriginal Community Controlled Organisations (ACCOs) that are not primary health care organisations but rather specialise in providing home-based and residential aged care services to Indigenous elders. These aged care ACCOs are similar to ACCHOs in that they are ‘an incorporated organisation initiated by the Aboriginal Community and governed by an Aboriginal Body elected by the Aboriginal Community’ (Nunkuwarrin Yunti of South Australia Inc., 2015).

This study aimed to explore the overarching principles, practices, enablers and challenges of Aboriginal community-controlled services that provide aged care tailored to the needs of older Indigenous peoples and examine the aged care planning activities and implementation actions that guided the integration of aged care service delivery within the ACCHO primary health care model.

## Method

This study was undertaken by the Centre of Research Excellence in Aboriginal Chronic Disease Knowledge Translation and Exchange (CREATE), an Indigenous-led centre of research excellence housed within the Wardliparingga Aboriginal Health Equity theme at the South Australian Health and Medical Research Institute and funded by the National Health and Medical Research Council of Australia. We used Indigenous methodologies that incorporated Indigenous leadership and governance, strength-based perspectives, Indigenous research methods, the privileging of Indigenous voices and centring of Indigenous knowledges (Rigney, 1999; Wilson 2008; Smith, 2012; Watson, 2014). The research team was predominantly Aboriginal and was led by an Aboriginal chief investigator, and yarning approaches were used during data collection. In addition, we used the research process as an opportunity for capacity-building Aboriginal workforce. Throughout the research process, we followed nine ethical principles for Aboriginal health research as co-designed with Indigenous communities in South Australia (e.g., partnership, respect, reciprocity, ownership) (South Australian Health and Medical Research Institute, 2014).

The CREATE Leadership Group, comprising predominantly Indigenous and also non-Indigenous nominated representatives from the ACCHO sector, provided governance for the research. The Leadership Group met twice-yearly and identified aged care as 1 of 10 priority domains for Indigenous peoples. We undertook a systematic review of the international literature to elaborate practices applied by both primary health care and aged care services to support the well-being of older Indigenous peoples (Davy *et al.*, 2016). The Leadership Group then recommended that the research team explore best practice principles and practices in culturally aligned aged care provided by Aboriginal community controlled services since ACCHO service delivery models are infrequently described within academic reports and publications.

### Recruitment and data collection

The CREATE Leadership Group identified two exemplar services to explore and define approaches to best practice in Aboriginal community-controlled aged care:

1. Case Study 1: Aboriginal Community Services, a metropolitan ACCO *aged care service* in South Australia with a long history providing home-based, respite and residential aged care services;
2. Case Study 2: Institute for Urban Indigenous Health, a metropolitan ACCHO *primary health care service* in Queensland that had recently integrated home-based and respite aged care within the organisation’s primary health care service delivery model.

The research team approached each organisation inviting participation in an in-depth case study. During initial engagement and organisational approval processes, a Memorandum of Understanding was developed describing mutually agreed terms and conditions for the scope of work that included site visits, interviews and yarning circles with interested staff, and the completion of a Case Study Tool to collect descriptive information about the organisation. The research team subsequently visited the services to meet with staff and invite their voluntary participation in semi-structured interviews and/or yarning circles. During Case Study 2, a member of staff accepted an invitation to fulfil the role of an Aboriginal Research Fellow. In a two-way learning approach, the Aboriginal Research Fellow received support and capacity strengthening from the research team and provided the research team with expert insights and interpretations regarding aged care practice. The Aboriginal Research Fellow undertook interviews and analysis and contributed to the interpretation and reporting of results.

Data collection for Case Study 1 was undertaken during multiple sites visits in July 2016 by three Aboriginal members of the research team. For Case Study 2, site visits and data collection occurred during May and June 2017 with interviews and yarning circles undertaken by the Aboriginal Research Fellow, an Aboriginal researcher and a non-Indigenous member of the research team. During interviews and yarning circles, participants were asked about their current role, about how aged care services came about in the organisation (i.e. why, for whom, when, and what was crucial for ensuring its development/implementation), how aged care services currently work (i.e. how it has changed over time, what currently works well and for whom, what currently does not work so well, what improvements could be made, and what is crucial for its sustainability) and what best practice does or should look like in the ACCHO sector (i.e. what is needed to consistently provide best practice service delivery, what issues should be considered in relation to the development of a best practice service delivery framework for ACCHOs). Interviews and yarning circles were digitally recorded with consent, and participants were provided with an opportunity to review transcribed text.

### Analysis

A framework approach to analysis (Ritchie *et al.*, 2014) was undertaken in NVivo Pro (QSR International) using an *a priori* coding framework developed by the research team. Transcribed and de-identified text from interviews and yarning circles were coded according to seven broad categories within the analysis framework: the community and organisational context; aged care service planning, integration and provision; contributing factors necessary for implementation and sustainability of services; challenges associated with service provision; perceived outcomes of service provision; recommendations for maintaining and expanding services; and implications for other ACCHOs considering aged care implementation and provision. Relevant contextual information sourced from the case study sites’ websites and 2014/2015 annual reports was used in the analysis.

Coding was undertaken by an Aboriginal researcher, the Aboriginal Research Fellow and a non-Indigenous researcher. Three Aboriginal researchers, the Aboriginal Research Fellow and two non-Indigenous researchers contributed to the analysis of coded data. Draft case study reports were compiled by the research team, reviewed and refined by key personnel within the case study sites prior to co-development of recommendations and finalisation of the reports. Key findings from the two case studies were synthesised, presented to and discussed with the CREATE Leadership Group during three face-to-face meetings and one teleconference. The Leadership Group contributed to the contextualisation of the findings for the ACCHO sector, offered additional insights from services in jurisdictions beyond the selected case study sites and highlighted challenges impacting Indigenous older peoples and the ACCHO sector more broadly.

## Results

Across the two case study sites, there were 46 members of ACCHO staff and Board of Management members who participated (Table 1). During Case Study 1, 18 members of staff participated in interviews, and during Case Study 2 there were 15 participant interviews, 3 group interviews (with two staff members in each) and one yarning circle with 7 participants. Data from Case Studies 1 and 2 was used to inform the principles, enablers, challenges and outcomes of Aboriginal community-controlled aged care, and data drawn from Case Study 2 informed the ways of working, aged care planning and implementation actions related to aged care service provision integrated within primary health care. The reported principles, enablers, challenges and perceived outcomes of Aboriginal community-controlled aged care are listed in Table 2 and described in detail below.

**Table 1.** Case study participants

Participants	Case study 1		Case study 2	
	Aboriginal peoples	Non-Indigenous peoples	Aboriginal peoples	Non-Indigenous peoples
Board Member and Executive Manager – non-clinical	2	2	3	2
Coordinator and Manager – clinical	1	6	–	2
Aged care worker and community workers	2	5	9	1
Health care worker	–	–	2	5
Transport worker	–	–	1	1
Finance	–	–	–	2
Totals	5	13	15	13

**Table 2.** Summary of principles, enablers, challenges and outcomes of Aboriginal community-controlled aged care service delivery

Principles	Enablers
• Respect traditional Aboriginal customs, values and beliefs • Support Aboriginal identity • Connect with elders and communities • Culturally safe care • Focus on holistic well-being • Respect self-determination • Tailored services • Willingness to go the extra mile • Maintain credibility	• Strong governance • Effective leadership • Organisational culture centred on respect for clients’ Aboriginal identity • Local, caring, qualified and culturally safe aged care workforce • Effective workforce recruitment and training processes • Effective communication • Clear referral pathways • Effective organisational structures and operating systems • Effective financial management systems • Continuous quality improvement processes • Relationships with external organisations
Challenges	Outcomes
• Funding challenges • Workforce shortages • Change management processes • The need to rapidly develop knowledge of the aged care system • Aged care reforms • Lack of coordination between government departments • Aged care eligibility requirements • Navigating the online aged care portal • Unclear correspondence from social services and government departments	• Promotion of Aboriginal identity and cultural connections • Increased number of elders receiving aged care • Reduced pressure and responsibilities on family carers • Improvements in physical health outcomes • Increased access to affordable, culturally safe and quality aged care • Reduced complexities associated with navigating multiple services • Economies of scale • Increased numbers of local, qualified, culturally safe aged care workers

### Principles

Guiding principles of Aboriginal community-controlled aged care service delivery were identified across the two aged care case studies. The identified principles highlighted how respect for culture, cultural identity, cultural safety and community connection are paramount to promote holistic (i.e., social, emotional, cultural, physical) well-being in aged care service delivery.

Staff members spoke of the significance of being an Aboriginal organisation which meant respecting traditional Aboriginal customs, values and beliefs and supporting the Aboriginal identity of clients. This underpinned all aspects of service delivery and was closely related to other principles. Connection with elders and communities was enabled by the employment of Indigenous staff who actively built and maintained relationships with elders, their families and communities to understand the unique needs of elders and facilitate connections.

*[I]t’s about them probably, knowing this is an Aboriginal organisation and Aboriginal people work here. They kind of feel connected to start with, they’re willing to have a go, to come out and have a go. [Aboriginal ACCO staff member]*

Participants described providing culturally safe care to older Indigenous peoples which was guided by strong Aboriginal governance and enabled through Indigenous workforce and cultural safety training for all staff.

*“Sorry, just to add to that, and I guess it is the, it’s the value of having Aboriginal workers and the value of having other Aboriginal elders - that’s a huge part of the feedback is, um, you know, it’s culturally safe and it’s, um, and I feel something familiar and, yeah, that sense of connectedness, I guess, that comes with that”. [Non-Indigenous ACCHO staff member]*

A focus on holistic well-being consistent with Aboriginal understandings of health was demonstrated through programs that supported the social, emotional, cultural and physical well-being of older Indigenous clients. The services provided opportunities for aged care clients to strengthen their Indigenous identity and practice culture as well as connect with other community members during social events, cultural celebrations and trips on country.

*[I]t’s about meeting other Aboriginal people that they know. It’s about being in a happy environment, it’s about sharing food, sharing stories. [Aboriginal ACCO staff member]*

Participants reflected respect for self-determination when describing client-directed care approaches where older Indigenous peoples were supported to make decisions about the nature of care they receive.

*Don’t make decisions for them, ask them. And I know that it’s – it’s talked about a lot, and it doesn’t happen often enough. I think that’s something people could learn from us. [non-Indigenous ACCO staff member]*

In both organisations, participants spoke of consulting with older Indigenous peoples and providing tailored services to meet the unique needs of their local communities.

*[A]s well as having the elders on our governing Board, our Board of management, we also have a client advisory group, that client advisory group for the Adelaide programs is made up of elders who receive our services and we meet three or four times a year just to talk about what we’re doing how well we’re doing it whether there are any things which we should be doing more of or changing and that’s direct feedback to us from some of the elders. [Aboriginal ACCO staff member]*

Individuals spoke of a strong personal commitment to work and managers spoke of employing staff with a fundamental compassion for older Indigenous peoples and a willingness to go the extra mile. Some participants, and in particular non-Indigenous staff members, reflected on the importance of maintaining credibility in the eyes of clients, client’s families and the broader community.

*Because that would be the worst thing, if I say, “I’ll get this all fixed and wave my magic wand,” and then I couldn’t do it, that client is not going to trust me any longer. They are going to think that, oh, yeah, she says she’ll do it and doesn’t. So I don’t. Unless I know damn well I can do it, I don’t ever say I can. I’ll just say, “I will try.” [non-Indigenous ACCO staff member]*

### Enablers

Many factors that contribute to effective Aboriginal community-controlled aged care service delivery were identified across the two case studies. Participants spoke of strong governance where the Board of the organisation provided integral connection to community, strategic planning, needs assessment and priority setting to inform the development of programs targeted to the needs of elders in the community.

*[H]aving a good mix on the Board is very important, a mixture of skills and experience and knowledge, clearly our Board has a very good mix in that respect, have a very good understanding of their role and responsibility and executing their fiduciary duties et cetera. What’s also important about our Board is that they have a very good relationship with the management team and also all of our staff, they feel part of the organisation and that relationship between the management and in particular myself and the Board is – is probably one of the strongest parts of the – the organisation’s operations. [Aboriginal ACCO staff member]*

Effective leadership was another identified enabler of aged care service provision across both aged care service providers.

*Yeah, it’s – it’s a hard one to define, but I think that it comes from the top. So, it comes from (CEO’s) philosophy. It comes from him wanting something better for the elders. [non-Indigenous ACCO staff member]*

An organisational culture centred on respect for clients’ Aboriginal identity was frequently reported as was the importance of a local, caring, qualified and culturally safe aged care workforce necessary for culturally safe aged care service provision. Effective workforce recruitment and training processes were considered important across both organisations where workforce shortages were commonplace.

*“So we then partnered with a training centre. We had to, and we developed the resources so they weren’t being trained in normal aged care, they were being trained in our aged care, so it was kind of contextualised and everything else. And as a result I’m thinking maybe, I’ve seen a few, about 60 something people have been trained up, um, since and the majority of those guys are in employment now”. [Aboriginal ACCHO staff member]*

Participants spoke of effective communication and clear referral pathways across the primary health care and aged care teams as positively contributing to service provision. Effective organisational structures and operating systems were a necessary enabler of aged service delivery, with effective financial management systems enabling the management of multiple income streams across aged care and primary health care services. Engaging with community through continuous quality improvement processes ensured that services were safe and tailored to the needs of older Indigenous clients. Finally, relationships with external organisations ensured that through effective advocacy, the holistic needs of older clients could be met, and workforce training needs could also be met through external providers where necessary.

### Ways of working: aged care integrated within the ACCHO

Participants described how integration of aged care within the ACCHO enabled older Indigenous clients to access both holistic primary health and aged care services through a continuous and coordinated care model. Care coordination was provided through the work of both the Primary Health Clinic and Aged Care Teams and managed through informal ‘yarns’ (an informal style of conversation) and formal multidisciplinary case conferences attended by the elder as well as key personnel such as Aged Care Workers, General Practitioners, Aboriginal Health Practitioners and Allied Health personnel.

**Figure 1.**
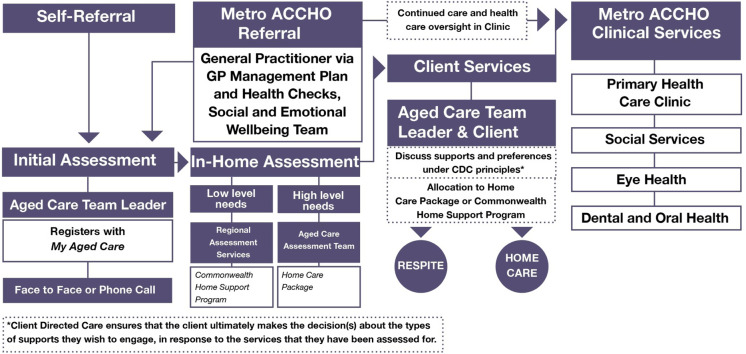
Referral pathways for aged care clients within the ACCHO

The ACCHO established referral pathways to their Aged Care Team that included self-referral, and referral from both the health clinic and community teams (e.g. the Social and Emotional Wellbeing Team). A practical model of the organisation’s aged care referral pathway is depicted in Figure 1.

The Aged Care Team spoke of supporting clients to navigate the aged care system and to get the most out of the funding available to them. This included registering for aged care services, accessing in-home assessments and yarning with clients to create an Aged Care Plan based on their needs and preferences. This could include home care services (such as personal care, clinical care, domestic assistance, home maintenance), respite care, social support and advocacy. A Community Visitor Scheme was also in place to connect with Indigenous older peoples living in both mainstream residential facilities and at home.

### Aged care planning

ACCHO staff members provided several examples of planning activities they had undertaken to determine the readiness of the organisation to integrate aged care with primary health care services. These included activities to identify community need, determine aged care service gaps, identify the current and potential aged care workforce and understand how other ACCHOs provided aged care. Various audits and internal scoping exercises were undertaken to determine the readiness of the organisation and financial viability of taking on aged care. A summary of the aged care planning activities undertaken by the ACCHO is provided in Table 3.

**Table 3.** Aged care planning activities undertaken by the ACCHO

Aged care planning activities	Description
Consultations with elders	Elders were consulted regarding their experiences accessing aged care services including the factors that support and challenge access.
Mapping existing aged care services	Information was collated on aged care service providers in the region including where they are located and the number of elders currently receiving services.
Mapping aged care workforce	The number of qualified aged care workers in the region was examined in addition to the number of community members providing unpaid aged care services as family members and carers
Visiting an ACCHO already providing aged care services	Services already providing aged care were consulted, including interstate services
Desktop audit	An audit was undertaken to identify elders already receiving services and those who are potentially eligible to receive aged care services. The patient management system was used to collate data on the numbers of elders, their locations and the services they access
Aged care scoping review	An aged care scoping review was undertaken that explored domains such as quality aged care service delivery, aged care policies and procedures, accreditation Standards and requirements, funding viability, data and reporting systems, and clinic readiness.

### Implementation actions

The integration of aged care with the ACCHO’s primary health care model was seen to be enabled by the recruitment of key personnel with experience and knowledge of the aged care system (including terminology, software and systems, funding and output measurement) who guided the aged care planning and integration processes including development of an Aged Care Master Plan. The analysis identified nine implementation actions, not necessarily sequential, that supported the integration of aged care services within the ACCHO, as detailed in Table 4.

**Table 4.** Implementation actions for the integration of aged care within the ACCHO

Implementation actions	Description
Action 1	The ACCHO informed the Commonwealth Government of an intention to take on aged care
Action 2	An Aged Care Project Officer was employed to develop an Aged Care Master Plan
Action 3	Aged Care Management structures were established such as an Aged Care Integration Project Group that met regularly to manage the integration process, and the Joint Management Committee that included Managers from the primary health care and aged care teams, and provided oversight and leadership in relation to efficiencies, economies of scale and systems.
Action 4	The ACCHO underwent accreditation to become an aged care provider
Action 5	The ACCHO developed partnerships to support the provision of aged care
Action 6	An Aged Care Workforce strategy was established
Action 7	Aged Care management software and systems were purchased
Action 8	Staff credentials and compliance requirements were embedded within the organisation’s Human Resource Management System
Action 9	Service delivery models were developed for aged care services (both home care and respite services) including, for example, referral pathways, reporting, case management and transport

### Challenges

To reduce barriers to access for older Indigenous clients, both case study sites strived to provide aged care services at no out of pocket cost, and as a result funding challenges were frequently reported by participants. During the process of integrating aged care services, the ACCHO went into financial deficit. The ACCHO also met with a great deal of complexity in coordinating multiple sources of funding (both primary health care and aged care funding streams) and in providing a range of unfunded services such as advocacy and support.

Participants from both organisations reported workforce shortages, particularly a shortage of Indigenous staff. A lack of existing workforce led the ACCHO to invest strongly in growing their own qualified culturally safe aged care workforce through partnering with training organisations and developing resources.

*I believe there’s – there’s lots of opportunity for us to continue to grow, and, that growth is really dependant and reliant on us growing our staff, our staffing numbers, so having the right people with the right skills available at the right time is particularly important for us as we move forward and continue to grow. [Aboriginal ACCO staff member]*

The challenge of change management processes was discussed in relation to integrating aged care within the ACCHO’s primary health care service delivery model (e.g. communicating with staff, establishing referral pathways). Related to this was the burden placed on ACCHO staff to rapidly develop their knowledge of the aged care system (e.g., processes, funding, terminology, applications and accreditation requirements) and to develop effective financial management systems to manage the increased complexity.

The government’s aged care reforms were also described as a challenge, as was a reported lack of coordination between government departments.

*“There seems to be a real lack of integration between government bodies, so Department of Health for instance allocates a package but it’s paid through the Department of Human Services, um, so if their data programs aren’t you know, connecting, then that can create issues at a service level, so for instance, um, just in the last month for some reason, no one can quite tell me why, a client was only paid part of her subsidy.” [non-Indigenous ACCHO staff member]*

ACCHO workforce also described that the eligibility requirements of the aged care system were challenging for elders, and particularly for those with non-Indigenous spouses whose aged care eligibility criteria were different. They referred to challenges elders face in navigating the online portal for aged care access, and the considerable time they invest in supporting elders to navigate the system. Finally, they spoke of needing to support clients to interpret and respond in a timely way to unclear correspondence from social services and government departments.

### Perceived outcomes

Participants felt that ACCOs have inherent benefits, including that their connection to elders and community means they can better tailor services to client needs and promote Aboriginal identity and cultural connections.

*“[H]e just lit up as soon as he saw a Nunga come in and we started talking about people that he knew, he was originally over from (regional town), all this sort of stuff and he knew all the (Aboriginal kinship group) mob from (name of place) so he really lit up”. [non-Indigenous ACCO staff member]*

Participants reported that they were able to increase the number of elders receiving aged care which enabled the service to reduce the pressures and responsibilities on family carers. They also reported that providing better support for elders’ social, emotional and cultural well-being led to improvements in physical health outcomes. In particular, they reported that they were able to increase access to affordable, culturally safe and quality aged care.

*“By using the integrated model of care, um, and by establishing the way that we have around putting the – the older – the elder, at the centre of all of their care, not just aged care, we’ve been able to do it cost free, so there’s no - no need for co-contribution for our elders, so that we’ve broken down whatever barriers we can, to access the services, you know.” [Aboriginal ACCHO staff member]*

Participants provided examples of how the integrated care model and close connections with elders ensured the changing needs of older clients were identified and safeguarded in an ongoing way, through additional supports and services.

*“…transport officers, they’re really good at communicating back to the team where, say, there are changes they can sense if there are difficulties in getting a person in the car and all of those sorts of things that come up”. [Aboriginal ACCHO staff member]*

ACCHO workforce reported that the integrated model enabled elders to access their local ACCHO for both aged care and holistic primary health care reducing the complexities associated with navigating multiple services. Integration of services also enabled optimal discharge planning following hospitalisations and efficient referrals between the clinic and aged care teams. Integration of services also created economies of scale that supported unfunded activities such as transport services for elders. Finally, the service invested in workforce strategies which lead to increased numbers of local qualified culturally safe aged care workers.

*“So really an important thing too about the social determinants stuff, you look at somewhere like (Place Name) where employment, unemployment rates are high, and particularly for our people, the vast majority of guys that come through the training were long-term unemployed”. [Aboriginal ACCHO staff member]*

## Discussion

The ACCHO sector has been supporting older Indigenous peoples with holistic primary health care over many decades following the establishment of the first Aboriginal community-controlled service in 1971 (Khoury, 2015). Culture is at the centre of all ACCHO primary health care service delivery (Harfield *et al.*, 2018) with culturally respectful care attributed to employment of Aboriginal staff, welcoming spaces, the integration of cultural protocols, a social view of health and strategies to promote access (Freeman *et al.*, 2014). This cultural safety is valued by Indigenous clients who also value the accessibility, holistic and diverse nature of ACCHO services (Gomersall *et al.*, 2017). The added benefit that ACCHOs bring as an aged care provider, as demonstrated through the case studies reported here, is their ability to promote elder well-being and protect elder safety through intimate knowledge of and connections to family and community. The integrated primary health and aged care model also promotes access by reducing the complexities elders face in navigating multiple services and systems. It is well recognized that the social determinants of health play a key role in shaping the life expectancy gap for Indigenous peoples (Commonwealth of Australia, 2013; AIHW, 2016). ACCHOs lead the way in providing holistic services to tackle social factors (Khoury, 2015), and those supported to provide aged care services will be in a strong position to manage the comorbidities and social complexities that many older Indigenous peoples’ face.

This study demonstrates that ACCHOs are ideally positioned to provide aged care services since they provide culturally safe client-centred care, promote the cultural identity and connectedness of older Aboriginal peoples, support self-determined decision-making and enable elders to remain independent for as long as they can. This is consistent with a review of international evidence that identified culturally safe care, maintaining Indigenous identity and promoting independence were key domains related to well-being for older Indigenous peoples (Davy *et al.*, 2016). A range of perceived benefits of Aboriginal community-controlled aged care were reported by participants in this study including increased access to affordable, culturally safe and quality aged care that strengthened cultural identity and connections and led to improvements in health outcomes. This provides evidence to support the recommendation of the *Royal Commission into Aged Care Quality and Safety* (Commonwealth of Australia, 2019: 190) that aged care for Indigenous older peoples should be integrated with ACCHO and ACCO services. Our findings suggest that this would increase access to aged care services for Indigenous elders and promote and support elders to navigate multiple health, social and aged care services. In order for the Aboriginal community-controlled sector to be successful, however, the challenges identified within this study related to the aged care system and to the process of integrating aged care must be considered. Concurrent investment must also be provided to strengthen the Indigenous aged care workforce and tackle workforce shortages.

The findings elaborated here highlight the strengths and successes of metropolitan Aboriginal community-controlled aged care models, much needed given that a majority (81.4%) of Indigenous peoples live in urban and regional areas (ABS, 2018). This adds to the existing evidence base on well-being and quality of life in older Aboriginal peoples (Radford *et al.*, 2014; Davy *et al.*, 2016; Smith *et al.*, 2017; Davy *et al.*, 2018; Smith *et al.*., 2021) and remote aged care service delivery for Aboriginal peoples (Smith *et al.*, 2010; LoGiudice *et al.*, 2012; Bell *et al.*, 2015). The elucidated aged care planning processes and implementation actions can inform an expansion of Aboriginal community-controlled aged care services to meet the growing numbers of older Indigenous peoples. Importantly, the findings elaborated here are likely to be of relevance to primary health care providers, academics and policy makers in Canada, the US and New Zealand in developing models of service delivery to better support their respective First Nations, Native American and Maori elders.

The strengths of the ACCHO sector have been recognized in the recent *National Agreement on Closing the Gap* which was signed by all Australian governments and a collective of ACCOs – the Coalition of Peaks (Australian Government, 2020). The Agreement provides a blueprint for closing the gap on Indigenous disadvantage in Australia. ‘Building the Community-Controlled Sector’ is the second of four priority reforms, with co-signatories stating under Item 43 that:

*‘the Parties acknowledge that Aboriginal and Torres Strait Islander community-controlled services are better for Aboriginal and Torres Strait Islander peoples, achieve better results, employ more Aboriginal and Torres Strait Islander peoples and are often preferred over mainstream services’. (* *Australian Government, 2020* *, 8).*

If the first target of the National Agreement – to ‘Close the Gap in life expectancy within a generation, by 2031’ (Australian Government, 2020: 17) – is to be realized, the integration of aged care service provision within ACCHO primary health care services must be adequately resourced and supported by aged care workforce strategies, capacity building initiatives and governance and operational support. The access challenges experienced by older Indigenous peoples (Australian Association of Gerontology, 2018) must also be addressed, and identified actions for service providers considered (Department of Health, 2019).

This study applied case study methodology to understand the principles and practices of Aboriginal community-controlled aged care service provision. As such, the findings provide best practice exemplars but may not represent the experiences of all services across the Aboriginal community-controlled sector. The findings may be useful for ACCHOs considering integrating aged care. Points of difference will depend on the ACCHOs size, context, workforce capacity and the needs of the elders they serve. The case studies were undertaken with metropolitan-based services, and there are likely to be unique challenges facing ACCHOs in regional and remote settings in relation to taking on aged care service provision and building a culturally safe aged care workforce. Future research could explore the aged care service requirements of Aboriginal and Torres Strait Islander communities in different settings (i.e., urban, regional, remote). A comparison of aged care service delivery characteristics and outcomes across Aboriginal community-controlled services compared with those of mainstream (i.e., government-run or privately owned) aged care services could also be explored in future studies.

## Conclusion

Culturally responsive and accessible aged care services must be developed to meet the unique and often complex needs of a growing number of Indigenous elders. The Aboriginal community-controlled sector is ideally positioned to provide tailored aged care and to promote connection between Indigenous elders and health and social services. Where stand-alone Aboriginal community-controlled aged care services are not available, the ACCHO sector must be adequately resourced and supported to integrate aged care and primary health care service delivery models. Concurrent investment in strategies to strengthen the Indigenous aged care workforce is necessary to promote culturally safe aged care and effective advocacy for Indigenous elders.
